# Advance in microRNA as a potential biomarker for early detection of pancreatic cancer

**DOI:** 10.1186/s40364-016-0074-3

**Published:** 2016-10-22

**Authors:** Jing Huang, Jianzhou Liu, Kevin Chen-Xiao, Xuemei Zhang, W. N. Paul Lee, Vay Liang W. Go, Gary Guishan Xiao

**Affiliations:** 1 School of Pharmaceutical Science and Technology, Dalian University of Technology, Dalian, 116024 China; 2 Harbor-University of California Los Angeles Research and Education Institute, UCLA School of Medicine, Torrance, CA 90502 USA; 3 Genomics and Functional Proteomics Laboratories, Creighton University Medical Center, Omaha, NE 68131 USA

**Keywords:** Early detection, Pancreatic cancer, Cancer stem cells, microRNAs, Signal transduction, Biomarker

## Abstract

Pancreatic cancer is characterized as a disease with low survival and high mortality because of no effective diagnostic and therapeutic strategies available in clinic. Conventional clinical diagnostic methods including serum markers and radiological imaging (CT, MRI, EUS, etc.) often fail to detect precancerous or early stage lesions. Development of effective biomarkers is unmet for reduction of mortality of pancreatic cancer. MicroRNAs (miRNAs) are a group of small non-protein-coding RNAs playing roles in regulation of cell physiology including tumorigenesis, apoptotic escape, proliferation, invasion, epithelial-mesenchymal transition (EMT), metastasis and chemoresistance. Various altered signaling pathways involving in molecular pathogenesis of pancreatic cancer are mediated by miRNAs as a role of either oncogenes or tumor suppressors. Among biomarkers developed including protein, metabolites, DNA, RNA, epigenetic mutation, miRNAs are superior because of its unique chemical property. Recent study suggests that miRNAs may be promising biomarkers used for early detection of pancreatic cancer. This review will update the progression made in early detection of pancreatic cancer.

## Background

Pancreatic cancer has an exceptionally low 5-year survival rate (<5 %) and high mortality rate, making it the fourth leading cause of cancer mortality in developed countries [1]. As it is particularly located in an inaccessible position of the abdomen leading the common clinical presentation, greater than 80 % of the affected patients were diagnosed when occurring locally advancing or metastasis [2]. Thus the early diagnosis of pancreatic cancer is the key for successful treatment of the disease, though it is rendered uneasy to accomplish resulted from the deficiency of early warning signs. Although much more research into biomarkers has been investigated, few biomarkers are proven to be effective used for early diagnosis of the disease [3]. Therefore, seeking novel biomarkers with higher sensitivity or specificity is still a challenge.

miRNAs are small non-protein-coding RNAs consisting of 18–24 nucleotides in length involving in regulating multiple gene expression by degrading target mRNAs or inhibiting translation at the post-transcriptional level, thereby regulating various neoplastic processes including cell proliferation, migration, invasion, survival, and metastasis [4]. Disregulation of miRNA plays an important role in the pathogenesis, diagnosis and therapy of pancreatic cancer [3]. Here, we update progression made in miRNA as early diagnostic/prognostic biomarkers for pancreatic cancer.

## Cancer diagnosis by imaging and biomarkers

Imaging technology has been widely applied as routine methods in diagnosis, therapy and prognosis of varied tumor types including pancreatic cancer. Although common imaging modalities consisting of computed tomography (CT), magnetic resonance imaging (MRI), endoscopic ultrasound (EUS), endoscopic retrograde cholangiopancreatography (ERCP) have been remarkably improved in their detection of pancreatic tumor as by positron emission tomography (PET) or their combination, sensitivity and specificity of detection of pancreatic cancer at its early stage remains challenge [5, 6].

As for CA 19-9, widely used biomarker, fails to distinguish pancreatic cancer from benign pancreatic diseases and multiple carcinoma, which limits clinical utility because of its inadequate sensitivity and specificity [7]. Similarly, several other serum and tissue-based biomarkers (Table 1) suffering the same limitation as CA 19-9, thus none of them have good clinical utility for early diagnosis of pancreatic cancer [7]. Effective biomarkers thus coupled with imaging would provide an ideal approach for early detection of cancer [5].

**Table 1:** Assay parameters of imaging modalities and classical biomarkers used in detection of pancreatic cancer

Item	AUC	Sensitivity (%)	Specificity (%)	PPV (%)	NPV (%)	Accuracy (%)	Reference
CT	0.832	92	-	80	67	-	[19]
MRI	0.92	100	-	90	100	-	[19]
EUS-FNA	-	82.1	100	100	79.2	89.4	[20]
MRCP	-	-	-	85	-	80	[21]
ERCP	-	-	-	88	-	85	[21]
MRI+ERCP	-	-	-	91	-	88	[21]
^18^F-FDGPE/CT	0.759	67.50	72.73	94.74	23.53	68.13	[22]
CA19-9	0.857	75.00	81.82	96.77	31.03	75.82	[22]
^18^F-FDGPE/CT + CA19-9	0.940	96.25	63.64	95.06	70.00	92.31	[22]
CA125	0.810	78.68	71.05	79.63	51.92	-	[23]
CEA	0.670	63.24	63.16	75.44	48.98	-	
CA50	0.630	52.21	78.95	81.61	48.00	-	
CA724	0.670	65.44	68.42	78.76	52.53	-	
CA242	0.640	64.71	60.53	74.58	48.94	-	
AFP	0.490	43.38	61.84	67.05	37.90	-	

The assay parameters are for diagnosis of differentiation between adenocarcinoma and nonadenocarcinoma. Small liver metastases (0.5 – 1 cm in diameter) were missed on CT and MRI. Adequate specimens were obtained by EUS-FNAB from 47 of the 50 pancreatic lesions (94.0 %). CA125, CEA, CA50, CA50, CA724, CA242 and AFP are for patients with unresectable pancreatic cancer misjudged as resectable tumor by CT scan. *AUC* area under the curve, *NPV* negative predictive value, *PPV* positive predictive value, − No data was available in these instances

## MicroRNAs as effective biomarkers

Currently, a large sum of studies have identified the potential role of miRNAs in tumorigenesis and metastasis, suggesting that it may be developed as biomarkers used for diagnosis, prognosis and prediction of pancreatic cancer [8]. Given that miRNA-mediated transcriptional regulation is involved in every cellular process, abnormal alterations in miRNA expression are commonly associated with all the carcinogenic process of pancreatic ductal adenocarcinoma (PDAC), including apoptosis escape, proliferation, invasion, epithelial-mesenchymal transition (EMT), metastasis and chemoresistance [8, 9]. Based on their expression, a handful of miRNAs up-regulated in tumor cells are classified as potent oncogenes, while some others are classified as tumor suppressors since they are conversely down-regulated during the tumorigenesis [10]. MiR-21, for instance, is classified as an oncogene because whose over-expression is associated with the increased proliferation, invasion, and chemoresistance of pancreatic cancer cells to Gemcitabine. Like miR-21, several other miRNAs, which clearly presented their over-expression in cancer tissues, include miR-155, miR-106a, miR-27a, miR-221/222, miR-224, miR-486, miR-194, miR-200b/c, miR-429, miR-10a/b, miR-367, miR-196a/b, miR-210, miR-375, and miR-301a [9, 10]. Meanwhile, another group of miRNAs showed inhibitory effects on cell proliferation, invasion and metastasis, thus functioned as tumor suppressors, including miR-34a/b, Let-7, miR-96, miR-124, miR-615-5p, miR-200a/b/c, miR-219-1-3p, miR-203, miR-146a, and miR-17-92 [9, 10]. An interesting study showed that miR-17-92 cluster is down-regulated in pancreatic cancer stem cells (CSCs), which showed highly resistant to chemotherapy by activating NODAL/ACTIVIN/TGF-β1 signaling pathways, thus, suppression of these pathways may enhance the sensitivity of pancreatic CSCs to chemotherapy [11].

**Fig. 1:**
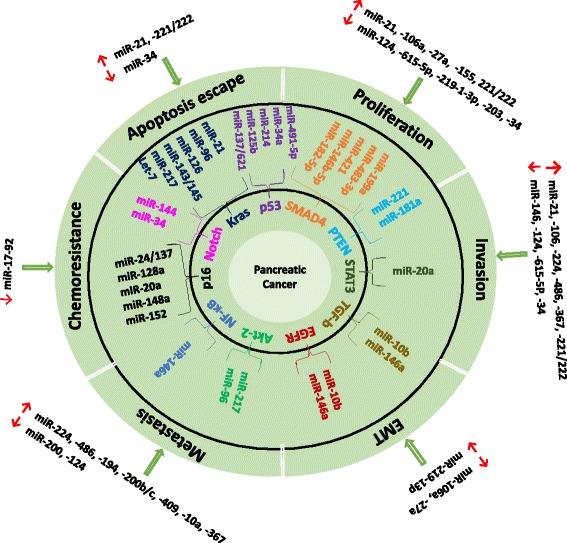
Potential miRNA biomarkers and their associated targeting pathways in pancreatic cancer. ↑ up-regulation ↓ down-regulation

miRNAs regulate key biological processes of tumor cells by altering signaling pathways involved in molecular pathogenesis of PDAC, including *K-ras*, p53, SMAD4, E-cad, PTEN, ADAM9, Bcl-2, STAT3, Cyclin D1, EGFR, TGF-β, JNK, Hedgehog, Notch, NF-κB, and Akt-2 [2]. RAS associated signaling pathways are derived from the most frequently mutated *KRAS* in 90 % of pancreatic cancer, whose translation was found to be mediated by miR-96, miR-126, miR-143/145, miR-217 and Let-7 [3]. Recently p53 has been shown to mediate regulation of miR-34 family (miR-34a/b/c). miRNAs such as miR-21, miR-143, miR-155, miR-130a/301a/454, miR-224, miR-146a, miR-483-3p, miR-494, miR-494, and miR-421 are involved in pancreatic cancer progression by regulating TGF-β/SMAD-4 signaling either directly or indirectly [3]. Moreover, miR-137 targets KDM4A mRNA during Ras-induced senescence, and activates both p53 and retinoblastoma (pRb) [12]. More information about miRNAs as biomarkers and their targeting pathways are summarized in Fig. 1.

As aberrant expression of miRNA occurs in molecular pathogenesis of pancreatic cancer, identification of miRNA signatures differentially expressed in different pathological stages of the disease may distinguish pancreatic cancer from pancreatic benign diseases with excellent sensitivity or specificity. A recent study in serum from patients with pancreatic cancer demonstrated that seven serum miRNAs, including miR-20a, miR-21, miR-24, miR-25, miR-99a, miR-185, miR-191, differentially expressed in pancreatic cancer compared to cancer-free subjects, suggesting this miRNA profile may be developed as an effective biomarker used for early detection of the disease with high sensitivity and specificity [13]. More study identified a panel of five miRNAs including miR-10b, −155, −106b, −30c, and −212 in plasma and bile had excellent accuracy, sensitivity, and specificity for detection of PDAC over the control(patients with choledocholithiasis but normal pancreata) [14]. Similarly, miR-21, miR-210, miR-155, and miR-196a showed significant difference in cancer versus healthy control, further study on miR-21 suggests that this miRNA can be developed as an independent prognostic biomarker distinguishing invasive from non-invasive macroscopic intraductal papillary mucinous neoplasms (IPMN), which is one of the precursor lesions of PDAC [15, 16]. It is worth noting that miRNA biomarkers can play a supplementary role with several protein markers in early identification of pancreatic cancer. The combination of a panel of miRNAs, especially miR-16 and miR-196a with CA19-9, for instance, showed effective at identification of tumors in Stage 1 [17]. Pancreatic cancer -initiating cell (PaCIC) markers, such as CD44v6, Tspan8, EpCAM, MET and CD104, combined with miRNA serum-exosome biomarkers including miR-1246, miR-4644, miR-3976 and miR-4306, improved sensitivity significantly with a specificity of 80 % for pancreatic cancer compared to all others groups [18]. Table 2 lists the sensitivity and the specificity of the miRNAs mentioned above.

**Table 2:** The AUC, sensitivity and specificity of miRNA-based biomarkers for detection of pancreatic cancer

*Item*	*Drived from*	*AUC*		*Sensitivity (%)*		*Specificity (%)*		*Reference*
		PCa *vs.* Normal	PCa *vs.* CP	PCa *vs.* Normal	PCa *vs.* CP	PCa *vs.* Normal	PCa *vs.* CP	
miR-20a, 21, 24, 25, 99a, 185, 191	Serum	0.992		89		100		[13]
miR-10b, 155, 106b, 30c, 212	Plasma	>0.90		95		100		[14]
	Bile			96		100		
miR-21, 210, 155, 196a	Plasma	0.82		64		89		[15]
CA19-9	Serum	0.903	0.897	81.2	81.2	100.0	89.0	[17]
miR-16, 196a,	Plasma	0.895	0.790	87.0	75.4	73.5	66.4	
miR-16+miR-196a+CA19-9	-	0.979	0.956	92.0	88.4	95.6	96.3	
PaCIC marker	Serum	-		0.96		0.86		[18]
miR-1246, 4644, 3976, 4306	Serum	-		0.81		0.94		
PaCIC marker + miR-1246, 4644, 3976, 4306	Serum	-		1.00		0.80		

Serum miR-20a, 21, 24, 25, 99a, 185 and 191 were derived from 197 PCa cases and 158 age- and sex-matched cancer-free controls. MiR-10b, 155, 106b, 30c and 212 were derived from patients (*n* = 215) with treatment-naive PDAC (*n* = 77), CP with bile/pancreatic duct pathology (*n* = 67), and controls (*n* = 71). The plasma levels of miR-21, 210, 155, 196a were interrogated in 49 PCa and 36 normal healthy individuals. Plasma miR-16, 196a and CA19-9 were extracted from 140 PCa patients, 111 CP patients and 68 normal controls. PaCIC marker and miR-1246, 4644, 3976, 4306 were collected from 20 healthy donors, 131 PCa, 25 CP, 22 benign pancreatic tumors, 12 nonPCa. *PCa* pancreatic cancer, *CP* chronic pancreatitis, *AUC* area under the curve, − No data was available in the instances

## Future perspective

Although more data suggest that miRNAs offers great potential as biomarkers for early detection of pancreatic cancer, there are limited prospective validation studies to prove their efficacy. Most studies thus far have been in the case-control stage, its application in clinic for prediction and diagnosis of pancreatic cancer at its early stages remained challenge. This may be due to lack of effective standard operational procedures (SOPs) used in standardized clinical assays. Secondarily, no consensus is agreed about the mechanisms underlying miRNA deregulation in tumor cells, hence understanding better the role of miRNAs in tumorigensis may eradicate the field of both molecular diagnosis and effective therapy of pancreatic cancer.

## Conclusions

As gene regulators, miRNAs can regulate cell growth, differentiation and apoptosis in many cases of human tumor. Abnormal expression of miRNAs in pancreatic cancer is the early events of pancreatic cancer development, which makes it can be used as a new biological marker for early detection of pancreatic cancer. Evaluation of single miRNA plays an important role in biomarker research, however, a single biomarker is often limitaed in sensitivity and specificity. A miRNA profile consists of a panel of up-regulated or down-regulated miRNAs, thus it can reflect the tumor progression with high sensitivity and specificity. Although there have been a large number of literatures which stated the potetial role of miRNAs as biomarker in pancreatic cnacer early detection, the application in clinical is still remains to explore.

## Abbreviations

CSCs: Cancer stem cells
CT: Computed tomography
EMT: Epithelial-mesenchymal transition
ERCP: Endoscopic retrograde cholangiopancreatography
EUS-FNA: Endoscopic ultrasound-Fine needle aspiration
IPMN: Intraductal papillary mucinous neoplasms
MRI: Magnetic resonance imaging
PaCIC: Pancreatic cancer-initiating cell
PDAC: Pancreatic ductal adenocarcinoma
PET: Positron emission tomography
pRb: p53 and retinoblastoma
SOPs: Standard operational procedures
